# Prevalence and predictors of Anxiety and Depression among Adolescents and Young Adults: Findings from the MoreGoodDays Support Program in Alberta, Canada

**DOI:** 10.1192/j.eurpsy.2024.251

**Published:** 2024-08-27

**Authors:** A. Belinda, R. Shalaby, K. Hay, R. Pattison, E. Eboreime, M. Korthuis, Y. Wei, V. I. O. Agyapong

**Affiliations:** ^1^University of Alberta; ^2^Kickstand, Edmonton; ^3^Dalhousie University, Halifax; ^4^Glenrose Rehabilitation Hospital Foundation, Edmonton, Canada

## Abstract

**Introduction:**

The COVID-19 pandemic has led to a rise in psychological disorders among adolescents and young adults. There is an increase in the prevalence of likely anxiety and likely depression among the subscribers of MoreGoodDays supportive text message program, reflecting the impact of the COVID-19 pandemic on this cohort.

**Objectives:**

To assess the prevalence, severity, and correlates of likely generalized anxiety disorder (GAD) and likely major depressive disorder (MDD) among subscribers of MoreGoodDays program.

**Methods:**

This study used a cross-sectional design. An online survey questionnaire was used to collect sociodemographic and clinical information from subscribers of MoreGoodDays program, a daily supportive text message program co-designed with adolescents and young adults for their peers in Alberta. Validated instruments, the Generalized Anxiety Disorder GAD-7 and Patient Health Questionnaire-9 PHQ-9 were used to collect information on likely GAD and likely major depressive disorder (MDD), respectively. Data was analyzed with SPSS version 25 using chi-squared tests and binary logistic regression analysis.

**Results:**

343 subscribers of MoreGoodDays participated in the survey. Overall, 117 (56.0%) respondents had a likely MDD and 97 (46.6%) had a likely GAD. Participants who would like to receive mental health counselling were 27 times more likely to experience GAD (OR = 27; 95% CI: 3.09–250.00) and 40 times more likely to experience MDD (OR = 40.03; 95% CI: 4.43–361.51) than those who did not. Respondents who had received mental health counselling in the past were 18.5 times more likely to experience MDD compared with those who had not (OR = 18.52; 95% CI: 1.55–200.00). Demographic variables, including age, education, employment, and relationship status, and clinical variables, such as history of anxiety, depression, obsessive-compulsive disorder, ADHD, and adverse childhood experience, did not independently the predict presence of likely GAD or MDD in subscribers of MoreGoodDays.

**Conclusions:**

The prevalence of anxiety and depression was relatively high among subscribers of MoreGoodDays, indicating the long-term effect of the COVID-19 pandemic. This finding has significant implications in the broader context of mental health research and emphasizes the need for more research into innovative mental health support for this cohort. The desire to receive counselling was predictive of both anxiety and depression and is a positive sign of the openness of this cohort to receive psychological intervention. Since this group is mostly adapted to mobile text technology, government agencies and policymakers should prioritize and implement readily accessible interventions such as supportive text messages to support their psychological well-being.

**Disclosure of Interest:**

None Declared

